# Is systems pharmacology ready to impact upon therapy development? A study on the cholesterol biosynthesis pathway

**DOI:** 10.1111/bph.14037

**Published:** 2017-11-26

**Authors:** Helen E Benson, Steven Watterson, Joanna L Sharman, Chido P Mpamhanga, Andrew Parton, Christopher Southan, Anthony J Harmar, Peter Ghazal

**Affiliations:** ^1^ Centre for Integrative Physiology University of Edinburgh Edinburgh UK; ^2^ Northern Ireland Centre for Stratified Medicine University of Ulster, C‐Tric Derry UK; ^3^ Centre for Cardiovascular Science University of Edinburgh, The Queen's Medical Research Institute Edinburgh UK; ^4^ Division of Infection and Pathway Medicine University of Edinburgh Medical School Edinburgh UK; ^5^ Centre for Synthetic and Systems Biology CH Waddington Building, King's Buildings Edinburgh UK

## Abstract

**Background and Purpose:**

An ever‐growing wealth of information on current drugs and their pharmacological effects is available from online databases. As our understanding of systems biology increases, we have the opportunity to predict, model and quantify how drug combinations can be introduced that outperform conventional single‐drug therapies. Here, we explore the feasibility of such systems pharmacology approaches with an analysis of the mevalonate branch of the cholesterol biosynthesis pathway.

**Experimental Approach:**

Using open online resources, we assembled a computational model of the mevalonate pathway and compiled a set of inhibitors directed against targets in this pathway. We used computational optimization to identify combination and dose options that show not only maximal efficacy of inhibition on the cholesterol producing branch but also minimal impact on the geranylation branch, known to mediate the side effects of pharmaceutical treatment.

**Key Results:**

We describe serious impediments to systems pharmacology studies arising from limitations in the data, incomplete coverage and inconsistent reporting. By curating a more complete dataset, we demonstrate the utility of computational optimization for identifying multi‐drug treatments with high efficacy and minimal off‐target effects.

**Conclusion and Implications:**

We suggest solutions that facilitate systems pharmacology studies, based on the introduction of standards for data capture that increase the power of experimental data. We propose a systems pharmacology workflow for the refinement of data and the generation of future therapeutic hypotheses.

**Abbreviations**
API: Application Programme Interface
BPS: British Pharmacological Society
BRENDA: Braunschweig Enzyme Database
CID: compound identifier
FDA: US Food and Drug Administration
FDFT1: farnesyl‐diphosphate farnesyl transferase 1
GtoPdb: Guide to Pharmacology Database
HMGCR: hydroxymethylglutaryl‐coa reductase
HMGCS1: hydroxymethylglutaryl‐CoA synthase
HPC: high‐performance computing
KEGG: Kyoto Encyclopedia of Genes and Genomes
IUBMB: International Union of Biochemistry and Molecular Biology
IUPHAR: International Union of Basic and Clinical Pharmacology
n2s: name‐to‐structure
ODE: ordinary differential equation
SBGN: Systems Biology Graphical Notation
SBGN‐ML: Systems Biology Graphical Notation Markup Language
SBML: Systems Biology Markup Language

## Introduction

The expansion of available genomic and proteomic data has enhanced our understanding of biomolecular interaction networks. Consequently, the development of systems biology approaches has enabled us to better understand how cellular behaviour emerges from these networks (Boran and Iyengar, 2010a). Systems‐level approaches have been used to predict the on‐ and off‐target impacts of an intervention (Boran and Iyengar, 2010b) and to identify the most sensitive components in pathways that suggest candidate drug targets (Benson *et al.,* 2013). They also have the untapped potential to suggest therapies comprising combinations of drugs chosen to strategically reprogram biomolecular interaction networks in order to drive the system from a diseased to a healthy state (Zhao *et al.,* 2013; van Hasselt and van der Graaf, 2015; Watterson and Ghazal, 2010). This approach, known as *systems pharmacology* (Boran and Iyengar, 2010b; Westerhoff *et al.,* 2015), is underpinned by the expansion in pathway, pharmacology and medicinal chemistry databases.

For example, WikiPathways held 804 human pathways (http://www.wikipathways.org/index.php/WikiPathways:Statistics) with 253 added in 2015 (Kutmon *et al.,* 2016). Kyoto Encyclopedia of Genes and Genomes (KEGG) PATHWAY holds 518 pathway maps (Kanehisa *et al.,* 2017) (http://www.kegg.jp/kegg/docs/statistics.html). Reactome currently holds 2148 human pathways involving 10 684 proteins and isoforms (http://reactome.org/stats.html) (Croft *et al.,* 2014; Fabregat *et al.,* 2016). ChEMBL version 23 (Gaulton *et al.,* 2016) includes 14 675 320 bioactivities, and the International Union of Basic and Clinical Pharmacology/British Pharmacological Society (IUPHAR/BPS) Guide to Pharmacology (GtoPdb) contains 15 281 curated interactions in its 2017.5 release (Southan *et al.,* 2016). In 2016, the Food and Drug Administration (FDA) new drug approvals fell to 22, following 45 approvals in 2015 (US Food and Drug Administration, 2016a; US Food and Drug Administration, 2016bb). According to DrugBank release 5.0, their distinct molecular count of approved small‐molecule drugs is 2037 (Law *et al.,* 2013).

As this catalogue of pharmacological interactions grows and our understanding of pathway systems expands, it will be advantageous to integrate these resources in order to devise new potential therapies. Drug combination‐based interventions represent an opportunity for therapy development that can yield one‐size‐fits‐all or personalized/stratified therapies, and they can target pathways precisely rather than perturbing entire networks. Two US National Institute for Health workshop white papers have made a strong case for systems pharmacology (Sorger *et al.,* 2011) as a way to reduce attrition in therapy, to stimulate drug development, to bridge the gap between network biology and translational medicine and to enhance industrial–academic collaborations. Systems pharmacology is also likely to impact upon genomic medicine (Westerhoff *et al.,* 2015), Systems Pathology, Systems Biology and Pharmacometrics (van der Greef and McBurney, 2005; Vicini and van der Graaf, 2013) and the tools that could contribute to systems pharmacology have been described (Lehár *et al.,* 2007; Berger and Iyengar, 2009; Kell and Goodacre, 2014).

Previous work under the domain of systems pharmacology has primarily focussed on pharmacokinetic–pharmacodynamic modelling (Darwich *et al.,* 2017). Industry has evaluated systems pharmacology as a tool to inform trial design in areas of cardiovascular disease, endocrinology, neurogenerative disease, respiratory disease, oncology and infectious disease (Visser *et al.,* 2014) and to inform regulatory development (Visser *et al.,* 2014; Peterson and Riggs, 2015). There have a been a number of specific studies of nerve growth factor (Benson *et al.,* 2013), coagulation (Wajima *et al.,* 2009), innate immunity (Madrasi *et al.,* 2014), cancer (Abaan *et al.,* 2013) and atherosclerosis (Pichardo–Almarza *et al.,* 2015).

However, whilst there is much enthusiasm for systems pharmacology as a tool to improve the efficacy and safety of the drug development pipeline (van der Graaf and Benson, 2011; Rostami‐Hodjegan, 2012; Trame *et al.,* 2016), the practical challenges of systematically amalgamating pharmacology and pathway biology in a coherent framework have not been adequately addressed.

Here, we describe a systems pharmacology study of the cholesterol biosynthesis pathway, detailing the barriers to progress that we encountered and suggesting solutions to these impediments, before proposing a model of how systems pharmacology studies could be conducted in future. In particular, we build a dynamic ordinary differential equation (ODE) model of the pathway, which we parameterize as far as possible from the literature. We identify relevant pharmacological agents that act on this pathway and parameterize them as far as possible from the literature and online databases. We then use computational optimization techniques to identify the drug combinations that are most effective at suppressing the outputs of the pathway that lead to cholesterol production and that minimize off‐target effects. In completing our analysis, we identify many of the problems that prevent this type of work being undertaken routinely, and we suggest solutions that would enable systems pharmacology to make a regular contribution to therapy development.

As explored in previous studies (Mazein *et al.,* 2011; 2013; Watterson *et al.,* 2013; Bhattacharya *et al.,* 2014; Caspi *et al.,* 2016), the cholesterol biosynthesis pathway is critical to both cardiovascular health (Lewington *et al.,* 2007; Henderson *et al.,* 2016; Parton *et al.,* 2016) and innate immunity (Blanc *et al.,* 2011; Lu *et al.,* 2015; Robertson *et al.,* 2016). As the target of the statin class of drug, we would expect this pathway to be amongst the most thoroughly characterized, and for this reason, we have chosen it for our feasibility study of systems pharmacology. For simplicity, we have focused on the segment of the pathway that transforms acetyl‐CoA to squalene and that forks to produce geranylgeranyl‐diphosphate. As a precursor to cholesterol, we would expect squalene synthesis to track cholesterol synthesis and so we use this as a proxy. The branch of the pathway that produces geranylgeranyl‐diphosphate has been shown to mediate both the innate immune response (Blanc *et al.,* 2011) and the myopathy side‐effects associated with statin treatment (Wagner *et al.,* 2011). Any intervention that demonstrates a minimal impact on this branch will avoid one of the significant side effects associated with standard cholesterol lowering treatments.

## Methods

### Pathway production

We started from the representations available in KEGG (Kanehisa *et al.,* 2014), MetaCyc (Caspi *et al.,* 2016) and the GtoPdb (Southan *et al.,* 2016) taking these resources to be representative of the community of online pathway databases. We reviewed the primary literature to establish the structure of the mevalonate portion of the cholesterol biosynthesis pathway, in particular the enzymes involved in the pathway, the reactions they catalyse, their subcellular localization, the species in which they were identified and any known isoforms.

Diagrams of the pathway were created using the Systems Biology Graphical Notation (SBGN) (Le Novère *et al.,* 2009), the yEd diagram software (yWorks GmbH, http://www.yworks.com/products/yed) and the SBGN‐ED add‐on to VANTED (Czauderna *et al.,* 2010). From these diagrams, we built kinetic models as systems of ODEs.

The ODE model of this pathway was built using Michaelis–Menten kinetics to describe each step except the interactions consuming isopentenyl diphosphate and producing geranylgeranyl diphosphate and pre‐squalene diphosphate. These steps were described using mass action kinetics in order to simplify the process of calculating the steady state of the model and hence the steady state behaviour of the pathway. Mass action kinetics were justified by the expectation that the pathway interactions would operate far from substrate saturation making the dynamics robust against small fluctuations in enzyme concentration. Mass action rate constants were calculated from the K_cat_, K_m_ and K_i_ parameters as described elsewhere (Watterson *et al.,* 2013) and enzyme concentrations were taken from experimentally measured values (Watterson *et al.,* 2013).

The pathway map and the associated mathematical model are available from the Supporting Information Files S1 and S2 as Systems Biology Graphical Notation Markup Language (SBGN‐ML) (Van Iersel *et al.,* 2012) and Systems Biology Markup Language (SBML) files (Hucka *et al.,* 2003) respectively.

### Pathway parameterization

We identified the kinetic parameters that quantify each reaction unambiguously (K_m_ and K_cat_) using the Braunschweig Enzyme Database (BRENDA) (Chang *et al.,* 2015) and verified the values described against those in the primary literature. In many instances, enzymes were associated with multiple kinetic parameter sets. We selected kinetic parameters based upon the following criteria: (i) specificity to the wild‐type enzyme in one of the three main mammalian model species: human, mouse or rat; (ii) sourced from a primary literature reference describing *in vivo* or *in vitro* experimental data as opposed to computationally derived structural modelling data; and (iii) sourced from a reference that could be accessed and therefore verified. For many enzymes, this yielded a range of values for each parameter, and where this was the case, we used the mean of the values obtained.

### Inhibitor list

Inhibitor compounds not already indexed in GtoPdb were identified for each reaction from ChEMBL and BRENDA, databases that we took to be representative of the community of target databases. We included a compound in our set if it met three criteria: (i) the enzyme used in the assay was wild‐type from one of the three main mammalian model species: human, mouse or rat; (ii) an experimentally determined reaction‐specific inhibition constant (K_i_) was reported; and (iii) the assay conditions were reported. Crucially, all data were checked against the primary literature references. Where this yielded a range of inhibition constants for nominally identical compounds, the most potent K_i_ values were used.

We verified the correct chemical structures of the inhibitors by cross‐referencing the original references against the online chemical databases PubChem (Kim *et al.,* 2016) and ChemSpider (Pence and Williams, 2010). The actual chemical structures of the marketed statin drugs were established by checking the FDA labels and the international non‐proprietary name‐assigned structures on the World Health Organization MedNet site (https://mednet‐communities.net/inn). Comparison of unique structural identifiers allowed us to identify duplicates within the ChEMBL, BRENDA and literature‐derived dataset, and to establish whether the chemical structure reported in a given reference matched the marketed drug or research compound structures.

Curated content describing the enzymes in this pathway, their substrates and small molecule inhibitors was used to consolidate and expand GtoPdb using the same approach and guidelines as described elsewhere (Pawson *et al.,* 2014). The enzymes, list of inhibitors and kinetic parameters are now all updated in the July 2016.3 release of GtoPdb.

### Hypothesis generation

We combined ODE kinetic models, the pathway parameters and the inhibitor parameters to create a model describing the dynamics of the mevalonate pathway. We sought to identify the drug combination that would best suppress the production of squalene as a precursor for cholesterol, but would also maintain production of geranylgeranyl‐diphosphate at the same levels as in the absence of any inhibitors, thereby eliminating a significant side‐effect of treatment. Firstly, we identified the steady‐state activity of the pathway in the absence of any inhibitors. Then we used computational optimization to identify the drug combination that, at steady state, minimized squalene production, but left geranylgeranyl diphosphate production the same as in the absence of inhibitors.

This was implemented using the Genetic Algorithm function available on Matlab (MathWorks, http://www.mathworks.com) in parallel with a population size of 200 and a function tolerance of 10^−6^. Matlab was chosen as the modelling platform for its flexibility, stability and comprehensive libraries. The genetic algorithm started with one instance of a set of drug concentrations where each drug was assigned a concentration equal to its K_i_. A 199 further instances of sets of drug concentrations were automatically generated from this instance by adding Gaussian noise to the concentration of each drug (with standard deviation 1, the default setting). These 200 instances comprised the first generation of candidate interventions. All instances of sets of concentrations were evaluated for their efficacy at suppressing squalene synthesis whilst maintaining geranylgeranyl diphosphate production. Two hundred new instances were created as a second generation of candidate interventions from the two most effective instances of the first generation and with the addition of Gaussian noise. The 200 new instances were then themselves evaluated with the two most effective instances used to generate a further 200 new instances, the third generation. This process was repeated until we arrived at instances from which no improvement in efficacy could be found for 20 consecutive generations, at which point we interpreted the best performing instance identified thus far as optimal.

### Nomenclature of targets and ligands

Key protein targets and ligands in this article are hyperlinked to corresponding entries in http://www.guidetopharmacology.org, the common portal for data from the IUPHAR/BPS Guide to PHARMACOLOGY (Southan *et al.,* 2016), and are permanently archived in the Concise Guide to PHARMACOLOGY 2015/16 (Alexander *et al.,* 2015).

## Results

### Pathway production

**Figure 1:**
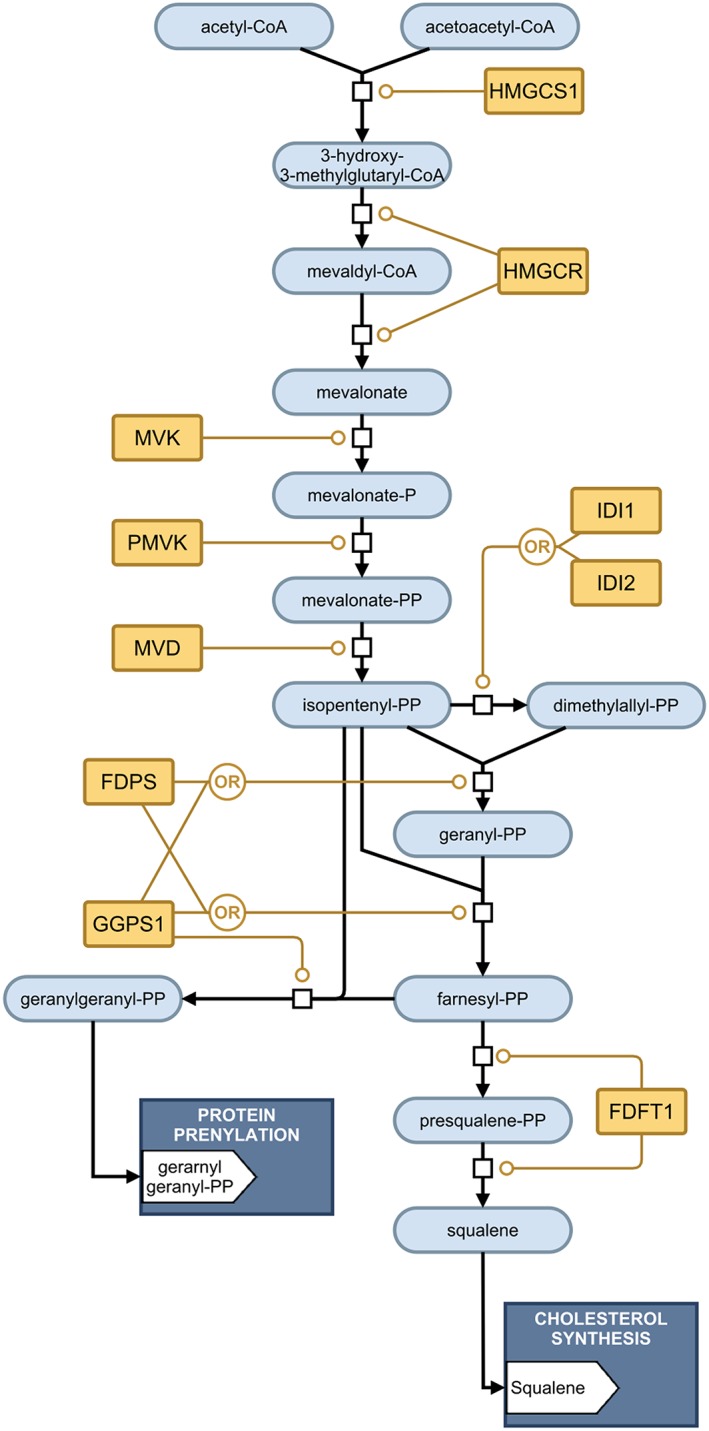
The mevalonate arm of the cholesterol biosynthesis pathway.

We produced the model of the mevalonate arm of the cholesterol biosynthesis pathway shown in Figure 1 in SBGN notation, describing the sequence of metabolic steps that lead from acetyl‐CoA and acetoacetyl‐CoA consumption to squalene and geranylgeranyl diphosphate production. This pathway comprises 12 steps (see Table 1), involving 10 enzymes and 14 metabolites.

**Table 1:** A list of the enzymes of the mevalonate branch of the cholesterol synthesis pathway, with gene and protein identifiers and EC numbers

E.C number	Enzyme/GtoPdb target ID	UniProt ID	HGNC ID	IUBMB enzyme approved name	Reaction catalysed	Km(mM)/PMID	Reported substrate/GtoPdb ligand ID	Kcat (s‐1)/PMID	Organism	Reported conditions	Mean Km (mM)	Substituted mean Km	Substituted mean Kcat
2.3.3.10	HMGCS1/638	Q01581	5007	Hydroxymethylglutaryl‐CoA synthase	Acetyl CoA + H_2_O + acetoacetyl CoA = (S)‐3‐hydroxy‐3‐methylglutaryl‐CoA + coenzyme A	0.009/6118268	Acetyl‐CoA/3038	–	Rattus norvegicus	absence of acetoacetyl‐CoA, hydrolysis reaction	0.0785	–	6.651575
	–	–	–	–	–	0.2/6118268	Acetyl‐CoA/3038	–	Rattus norvegicus	0.01 M acetoacetyl‐CoA	–	–	–
	–	–	–	–	–	0.073/19706283	Acetyl‐CoA/3038	–	Homo sapiens	–	–	–	–
	–	–	–	–	–	0.076/19706283	Acetyl‐CoA/3038	–	Homo sapiens	–	–	–	–
	–	–	–	–	–	0.084/19706283	Acetyl‐CoA/3038	–	Homo sapiens	–	–	–	–
	–	–	–	–	–	0.029/7913309	Acetyl‐CoA/3038	–	Homo sapiens	–	–	–	–
1.1.1.34	HMGCR/639	P04035	5006	Hydroxymethylglutaryl‐CoA reductase (NAPDH)	(S)‐3‐Hydroxy‐3‐methylglutaryl‐CoA + 2 NADPH = mevaldyl CoA + 2NADP^+^	0.006/4985697	3‐Hydroxy‐3‐methylglutaryl‐CoA/3040	–	Rattus norvegicus	Only one enantiomer	0.0765	–	0.0023
	–	–	–	–	–	0.012/4985697	3‐Hydroxy‐3‐methylglutaryl‐CoA/3040	–	Rattus norvegicus	–	–	–	–
	–	–	–	–	Mevaldyl CoA + 2NADP^+^ = (R)‐mevalonate + coenzyme A + 2 NADP^+^	0.01/10392455	3‐Hydroxy‐3‐methylglutaryl‐CoA/3040	–	Mus musculus	Enzyme from tumour	–	–	–
	–	–	–	–	–	0.014/10392455	3‐Hydroxy‐3‐methylglutaryl‐CoA/3040	–	Mus musculus	Enzyme from liver and tumour	–	–	–
	–	–	–	–	–	0.015/10392455	3‐Hydroxy‐3‐methylglutaryl‐CoA/3040	–	Mus musculus	Enzyme from liver, implanted tumour	–	–	–
	–	–	–	–	–	0.019/10392455	3‐Hydroxy‐3‐methylglutaryl‐CoA/3040	–	Mus musculus	Enzyme from liver, implanted tumour	–	–	–
	–	–	–	–	–	0.024/10392455	3‐Hydroxy‐3‐methylglutaryl‐CoA/3040	–	Mus musculus	Enzyme from liver	–	–	–
	–	–	–	–	–	0.07/16128575	3‐Hydroxy‐3‐methylglutaryl‐CoA/3040	–	Homo sapiens	–	–	–	–
	–	–	–	–	–	0.6/−−	3‐Hydroxy‐3‐methylglutaryl‐CoA/3040	–	Homo sapiens	pH 7.5/Temp not specified	–	–	–
	–	–	–	–	–	0.068/18446881	Hydroxymethylglutaryl‐CoA	0.023/18446881	Rattus norvegicus	–	–	–	–
	–	–	–	–	–	0.004/7077140	S‐3‐Hydroxy‐3‐methylglutaryl‐CoA/3040	–	Rattus norvegicus	–	–	–	–
2.7.1.36	MVK/640	Q03426	7530	Mevalonate kinase	ATP + (R)‐mevalonate = ADP + (R)‐5‐phosphomevalonate	0.035/14680942	(RS)‐mevalonate/3056	–	Rattus norvegicus	pH 7.5/25C	0.0337	–	–
	–	–	–	–	–	0.035/17964869	(RS)‐mevalonate/3056	21.9/18302342	Rattus norvegicus	pH 7.5/34C	–	–	–
	–	–	–	–	–	0.0408/18302342	(RS)‐mevalonate/3056	–	Homo sapiens	pH 7.5/30C	–	–	–
	–	–	–	–	–	0.024/9325256	Mevalonate/3056	–	Homo sapiens	pH 7.0/30C	–	–	–
2.7.4.2	PMVK/641	Q15126	9141	Phosphomevalonate kinase	ATP + (R)‐5‐phosphomevalonate = ADP + (R)‐5‐diphosphomevalonate	0.034/17902708	(R)‐5‐Phosphomevalonate/3046	–	Homo sapiens	pH 7.0/30C	0.034	–	6.651575
4.1.1.33	MVD/642	P53602	7529	Diphosphomevalonate decarboxylase	ATP + (R)‐5‐diphosphomevalonate = ADP + phosphate + isopentenyl diphosphate + CO_2_	0.02/8744421	5‐Diphosphomevalonate/3055	–	Rattus norvegicus	–	0.0262	–	–
	–	–	–	–	–	0.0289/18823933	5‐Diphosphomevalonate/3055	4.5/18823933	Homo sapiens	30C	–	–	–
	–	–	–	–	–	0.036/16626865	5‐Diphosphomevalonate/3055	–	Rattus norvegicus	–	–	–	–
	–	–	–	–	–	0.036/17888661	5‐Diphosphomevalonate/3055	–	Rattus norvegicus	–	–	–	–
	–	–	–	–	–	0.01/11913522	Mevalonate diphosphate/3055	–	Mus musculus	pH 7.2	–	–	–
5.3.3.2	IDI1 and IDI2*/646 & 647	Q13907/Q9BXS1	5387/23487	Isopentenyl‐diphosphate delta isomerase	Isopentenyl diphosphate = dimethylallyl diphosphate	0.0228/17202134	Isopentenyl diphosphate/3048	–	Homo sapiens	pH 8.0	0.0279	–	6.651575
	–	–	–	–	–	0.033/8806705	Isopentenyl diphosphate/3048	–	Homo sapiens	–	–	–	–
2.5.1.1	FDPS/644	P14324	3631	Farnesyl diphosphate synthase	Dimethylallyl diphosphate + isopentenyl diphosphate = diphosphate + geranyl diphosphate	–	–	–	–	–	–	0.0351375	6.651575
2.5.1.10	–	–	–	–	Geranyl diphosphate + isopentenyl diphosphate = diphosphate + trans,trans‐farnesyl diphosphate	–	–	–	–	–	–	0.0351375	6.651575
2.5.1.1	GGPS1/643	O95749	4249	Farnesyltranstransferase	Dimethylallyl diphosphate + isopentenyl diphosphate = diphosphate + geranyl diphosphate	–	–	–	–	–	–	0.0351375	6.651575
2.5.1.10	–	–	–	–	Geranyl diphosphate + isopentenyl diphosphate = diphosphate + trans,trans‐farnesyl diphosphate	–	–	–	–	–	–	0.0351375	6.651575
2.5.1.29	–	–	–	–	Trans,trans‐farnesyl diphosphate + isopentenyl diphosphate = diphosphate + geranylgeranyl diphosphate	0.0029/17846065	Isopentenyl diphosphate/3048	–	Rattus norvegicus	pH 7.0/37C	0.0027	–	–
	–	–	–	–	–	0.003/16698791	Isopentenyl diphosphate/3048	–	Homo sapiens	pH 7.7/37C	–	–	–
	–	–	–	–	–	0.00071/17846065	Trans,trans‐farnesyl diphosphate/3050	–	Rattus norvegicus	pH 7.0/37C	–	–	–
	–	–	–	–	–	0.0042/16698791	Trans,trans‐farnesyl diphosphate/3050	0.204/16698791	Homo sapiens	pH 7.7/37C	–	–	–
2.5.1.21	FDFT1/645	P37268	3629	Farnesyl‐diphosphate farnesyl transferase 1	2 Trans,trans‐farnesyl diphosphate = diphosphate + presqualene diphosphate	0.0023/9473303	Farnesyl diphosphate/2910	–	Homo sapiens	–	0.0016	–	6.651575
	–	–	–	–	Presqualene diphosphate + NAD(P)H + H^+^ = trans‐squalene + diphosphate + NAD(P)^+^	0.001/1569107	Trans‐farnesyl diphosphate/3050	–	Rattus norvegicus	–	–	–	–

Reported substrates, kinetic values and details of the experimental studies from which they were obtained, along with references are recorded.

Please note that ligands outlined in the table are listed using the nomenclature from the original literature. Where the reference did not specify the isomer used experimentally, it was assumed the racemate was used.

FDPS, farnesyl diphosphate synthase; IDI1, isopentenyl diphosphate delta isomerase 1; IDI2, isopentenyl diphosphate delta isomerase 2; MVD, diphosphomevalonate decarboxylase; MVK, mevalonate kinase; PMVK, phosphomevalonate kinase.

The parameters required for the resulting ODE model are shown in Table 1. After pooling results across mouse, human and rat models, we were able to obtain experimental values for only 12 out of the 24 required parameters. Across the studies reported, pH values ranged from 7.0 to 8.0 and temperatures ranged from 25°C to 37°C, although in some studies, neither pH nor temperature values were given. When verified against the primary references, we found that one parameter value obtained from BRENDA was missing from the literature reference provided, suggesting that it had been misattributed [K_cat_ = 0.023/s for **hydroxymethylglutaryl‐Coa reductase** (HMGCR)]. A second parameter had been transcribed (for diphosphomevalonate decarboxylase) where the literature source contradicted itself, specifying K_m_ = 10 μM in the abstract and K_m_ = 10 mM in the manuscript. Because computational hypothesis generation is highly sensitive to the values of the parameters, ambiguous or inaccurate reporting can have a significant impact on any predictions made.

Substrates were reported in varying levels of structural detail. Common names were used that could refer to multiple explicit forms of a chemical structure. However, variations in the chirality and chemical structure can significantly affect substrate affinity. The relative enzyme concentrations had been inferred previously (Watterson *et al.,* 2013) and are listed in Table 2.

**Table 2:** Normalized enzyme levels

Enzyme	Level
HMGCS1	1441
HMGCR	258
MVK	76
PMVK	874
MVD	111
IDI1	2707
IDI2	–
FDPS	7029
GGPS1	86
FDFT1	3425

FDPS, farnesyl diphosphate synthase; IDI1, isopentenyl diphosphate delta isomerase 1; IDI2, isopentenyl diphosphate delta isomerase 2; MVD, diphosphomevalonate decarboxylase; MVK, mevalonate kinase; PMVK, phosphomevalonate kinase.

Supporting Information Table S1 compares representations of the cholesterol biosynthesis pathway across the main publicly available pathway and chemical databases. It includes a summary of cross‐referencing between databases with standard identifiers for unambiguous representation, which will be essential for future cross‐platform interoperability.

### Inhibitors

The inhibitors obtained from GtoPdb, BRENDA and the literature, together with their inhibition constants (K_i_), are listed in Table 3. Six of the 10 enzyme targets had quantified parameters in humans. It was necessary to include two inhibitors that had been only reported for rat enzymes [L‐659,699 for **hydroxymethylglutaryl‐CoA synthase** (HMGCS1) and 3‐hydroxy‐3‐methyl‐6‐phosphohexanoic acid for phosphomevalonate kinase] in order to maximize coverage of the pathway. Two enzyme paralogues (isopentenyl diphosphate Δ‐isomerases 1 and 2) had no reported inhibitors with available K_i_ values, representing a region of the pathway that cannot currently be modulated in our modelling process. This can be contrasted with the enzymes HMGCR and farnesyl diphosphate synthase, each of which had an extensive list of inhibitors. Inhibition constants could be obtained for 8 of the 10 enzymes in the pathway. Where reported, these values came from studies conducted across a range of pH levels from 6.8 to 7.5 and temperatures from 25°C to 37°C.

**Table 3:** List of inhibitors for each of the enzymes in the mevalonate branch of the cholesterol synthesis pathway with K_i_ values and references

E.C number	Enzyme	Inhibitor name/GtoPdb ligand ID	InChi key	Approved drug?	Organism	K_i_ or IC_50_ (nM)/PMID	Reported conditions	Multidrug concentration (nM)
2.3.3.10	HMGCS1	L‐659699/5886	ODCZJZWSXPVLAW‐KXCGKLMDSA‐N	No	Rattus norvegicus	Ki = 53.7/7913309	–	0.215
1.1.1.34	HMGCR	Rosuvastatin/2954	BPRHUIZQVSMCRT‐YXWZHEERSA‐N	Yes	Homo sapiens	Ki = 2.3/16128575	pH 6.8, 37C	5.48
		Rosuvastatin/2954	BPRHUIZQVSMCRT‐YXWZHEERSA‐N	Yes	Homo sapiens	Ki = 3.5/12773150	–	–
		Rosuvastatin/2954	BPRHUIZQVSMCRT‐YXWZHEERSA‐N	Yes	Homo sapiens	Ki = 0.9/15686898	–	–
		Cerivastatin/2950	SEERZIQQUAZTOL‐ANMDKAQQSA‐N	Yes	Homo sapiens	Ki = 5.7/16128575	–	–
		Cerivastatin/2950	SEERZIQQUAZTOL‐ANMDKAQQSA‐N	Yes	Homo sapiens	Ki = 10/12773150	–	–
		Atorvastatin/2949	XUKUURHRXDUEBC‐KAYWLYCHSA‐N	Yes	Homo sapiens	Ki = 8/12773150	–	–
		Atorvastatin/2949	XUKUURHRXDUEBC‐KAYWLYCHSA‐N	Yes	Homo sapiens	Ki = 14/16128575	–	–
		Lovastatin/2739	PCZOHLXUXFIOCF‐BXMDZJJMSA‐N	Yes	Homo sapiens	Ki = 0.6/	doi: 10.1021/np50061a020	–
		Lovastatin/2739	PCZOHLXUXFIOCF‐BXMDZJJMSA‐N	Yes	Homo sapiens	Ki = 0.6/6933445	–	–
		Simvastatin/2955	RYMZZMVNJRMUDD‐HGQWONQESA‐N	Yes	Homo sapiens	Ki = 2.6/15686898	–	–
		CHEMBL39312/7991	VWKZOIOUHUHQKZ‐HZPDHXFCSA‐N	No	Homo sapiens	Ki = 3/	doi: 10.1016/S0960‐894X(01)80788‐5	–
		CHEMBL39102/7993	XKZCNQAYFRBCKR‐HNNXBMFYSA‐N	No	Homo sapiens	Ki = 16/	doi: 10.1016/S0960‐894X(01)80788‐5	–
		Fluvastatin/2951	FJLGEFLZQAZZCD‐MCBHFWOFSA‐N	Yes	Homo sapiens	Ki = 275/16128575	–	–
2.7.1.36	MVK	Farnesyl thiodiphosphate/3216	DRADWUUFBCYMDM‐UHFFFAOYSA‐L	No	Homo sapiens	Ki = 29/14679225	pH 7.5, 30C	0.0500
2.7.4.2	PMVK	Cinnamic acid/3203	WBYWAXJHAXSJNI‐VOTSOKGWSA‐N	No	Rattus norvegicus	Ki = 2480000/226078	pH 7.4, 37C	0.0240
		Isoferulic acid/7980**	QURCVMIEKCOAJU‐HWKANZROSA‐N	No	Rattus norvegicus	Ki = 3850000/226078	pH 7.4, 37C	–
		3‐hydroxy‐3‐methyl‐6‐phosphohexanoic acid/3202	XRCIRZGXKWCWNQ‐UHFFFAOYSA‐N	No	Rattus norvegicus	Ki = 145000/	doi: 10.1021/ja00493a044	–
		P‐coumaric acid/5787	NGSWKAQJJWESNS‐ZZXKWVIFSA‐N	No	Rattus norvegicus	Ki = 2390000/226078	pH 7.4, 37C	–
4.1.1.33	MVD	6‐fluoromevalonate 5‐diphosphate/3205	GLNCOGHKIHKSA‐UHFFFAOYSA‐N	No	Homo sapiens	Ki = 62/18823933	pH 7.0, 30C	0.0500
		2‐fluoromevalonate 5‐diphosphate/3204	WPXHWHACORBSDS‐UHFFFAOYSA‐N	No	Rattus norvegicus	Ki = 3020/16626865	pH 7.5, 25C	–
		Diphosphoglycolyl proline/3206	CDFDGXYBANXCPC‐UHFFFAOYSA‐N	No	Homo sapiens	Ki = 2300/18823933	–	–
		CHEMBL1160330/7994	YERUUUBBRAPJND‐UHFFFAOYSA‐N	No	Homo sapiens	Ki = 750/	doi: 10.1016/0960‐894X(96)00374‐5	–
		CHEMBL1160328/7996	YIGLDWRZXXHIGZ‐ZCFIWIBFSA‐N	No	Homo sapiens	Ki = 37/	doi: 10.1016/0960‐894X(96)00374‐5	–
		P′‐geranyl 2‐fluoromevalonate 5‐diphosphate/3207	ACYPMTKDKJZHBJ‐MDWZMJQESA‐N	No	Homo sapiens	Ki = 4169/18823933	–	–
		P′‐geranyl 3,5,9‐trihydroxy‐3‐methylnonanate 9‐diphosphate/5621	PMUQIJKCGIYWGT‐GZTJUZNOSA‐N	No	Rattus norvegicus	Ki = 6457/16524256	–	–
5.3.3.2	IDI1	–	–	–	–	–	–	–
5.3.3.2	IDI2	–	–	–	–	–	–	–
2.5.1.1, 2.5.1.10	FDPS	Zoledronic acid/3177	XRASPMIURGNCCH‐UHFFFAOYSA‐N	Yes	Homo sapiens	Ki = 0.07/18327899	–	12.7
		Zoledronic acid/3177	VWKZOIOUHUHQKZ‐HZPDHXFCSA‐N	Yes	Homo sapiens	Ki = 85/18327899	–	–
		Risedronate/3176	IIDJRNMFWXDHID‐UHFFFAOYSA‐N	Yes	Homo sapiens	Ki = 0.36/18327899	–	–
		Risedronate/3176	IIDJRNMFWXDHID‐UHFFFAOYSA‐N	Yes	Homo sapiens	Ki = 81/18327899	–	–
		NE58062/3166	XUCBNFJYKWKAMN‐UHFFFAOYSA‐N	No	Homo sapiens	Ki = 1/18327899	–	–
		NE97220/3171	NAIJOBGUXRHQJW‐UHFFFAOYSA‐N	No	Homo sapiens	Ki = 1.09/18327899	–	–
		NE97220/3171	NAIJOBGUXRHQJW‐UHFFFAOYSA‐N	No	Homo sapiens	Ki = 12/18327899	–	–
		NE58018/3168	XXNASZAYANFLID‐UHFFFAOYSA‐N	No	Homo sapiens	Ki = 0.74/18327899	–	–
		NE58018/3168	XXNASZAYANFLID‐UHFFFAOYSA‐N	No	Homo sapiens	Ki = 59/18327899	–	–
2.5.1.1, 2.5.1.10, 2.5.1.29	GGPS1	BPH‐628/3188	MPBUFKZCEBTBSK‐UHFFFAOYSA‐N	No	Homo sapiens	Ki = 20/17535895	–	13.5
		BPH‐608/7977	YXQQNSYZOQHKHD‐UHFFFAOYSA‐N	No	Homo sapiens	Ki = 60/17535895	–	–
		BPH‐675/7975	MZVWVRVNMXTDAK‐UHFFFAOYSA‐N	No	Homo sapiens	Ki = 70/17535895	–	–
		BPH‐629/7976	BYVXAUZOTGITQZ‐UHFFFAOYSA‐N	No	Homo sapiens	Ki = 110/17535895	–	–
		BPH‐676/7978	NWIARQRYIRVYCM‐UHFFFAOYSA‐N	No	Homo sapiens	Ki = 110/17535895	–	–
2.5.1.21	FDFT1	Zaragozic acid A/3057	DFKDOZMCHOGOBR‐NCSQYGPNSA‐N	No	Homo sapiens	Ki = 0.25/7864626	pH 7.4, 37C	4.79
		CHEMBL24362/3105	FBPJEWKDFUWVKV‐UHFFFAOYSA‐N	No	Homo sapiens	Ki = 43/	doi: 10.1016/S0960‐894X(97)00053‐X	–
		CHEMBL1208103/3120	HGDWHTASNMRJMP‐UHFFFAOYSA‐N	No	Homo sapiens	Ki = 300/19456099	Recombinant enzyme expressed in *Escherichia coli*	–
		CHEMBL1207858/3127	AGJZDRXKAQZWEP‐UHFFFAOYSA‐N	No	Homo sapiens	Ki = 520/19456099	Recombinant enzyme expressed in *E. coli*	–
		BPH‐830/3121	GNETVUVZFYJATO‐UHFFFAOYSA‐N	No	Homo sapiens	Ki = 530/19456099	Recombinant enzyme expressed in *E. coli*	–
		SQ‐109/7997	JFIBVDBTCDTBRH‐REZTVBANSA‐N	No	Homo sapiens	Ki = 740/22486710	Recombinant enzyme expressed in *E. coli*	–
		[1‐(Hydroxycarbamoyl)‐4‐(3‐phenoxyphenyl)butyl]phosphonate/3120	HGDWHTASNMRJMP‐UHFFFAOYSA‐N	No	Homo sapiens	Ki = 302/19456099	–	–
		Compound 13 [PMID: 19 456 099]/3127	AGJZDRXKAQZWEP‐UHFFFAOYSA‐N	No	Homo sapiens	Ki = 525/19456099	–	–
		(1‐Methyl‐1‐{[3‐(3‐phenoxyphenyl)propyl]carbamoyl}ethyl)phosphonate/3127	AGJZDRXKAQZWEP‐UHFFFAOYSA‐N	No	Homo sapiens	Ki = 525/19456099	–	–

** Denotes interaction not listed on GtoPdb. These reactions were selected from either BRENDA or ChEMBL to complete the dataset required for the modelling process.

FDPS, farnesyl diphosphate synthase; IDI1, isopentenyl diphosphate delta isomerase 1; IDI2, isopentenyl diphosphate delta isomerase 2; MVD, diphosphomevalonate decarboxylase; MVK, mevalonate kinase; PMVK, phosphomevalonate kinase.

Both explicit structure and name‐to‐structure (n2s) ambiguities existed around the reporting of inhibitor entities. In some cases, the common or trade name of a compound was used, without specification of the exact chemical structure and stereochemistry. In other cases, we found a different n2s assignment across different database resources or indeed within the same resource. For example, under the HMGCS1 entry of BRENDA, the same inhibitor is listed twice as L‐659,699 and (E,E)‐11‐[3‐(hydroxymethyl)‐4‐oxo‐2‐oxytanyl]‐3,5,7‐trimethyl‐2,4‐undecadienenoic acid.

Several results recorded in ChEMBL were transcribed against the incorrect drug target. Three inhibitors listed against the enzyme HMGCS1 describe results obtained from experiments with HMGCR (Balasubramanian *et al.,* 1989). There were also cases where the incorrect species had been recorded. For example, the compound with ChEMBL ID CHEMBL88601 cited in one study (Procopiou *et al.,* 1994) (ChEMBL document ID CHEMBL1151052) is listed against the human squalene synthase (FDFT1) enzyme, whilst in fact, the paper describes results for the yeast Candida albicans and rats.

### Hypothesis generation

In order to complete the gaps in the available parameter sets, we proceeded by assuming that where parameters were taken from separate studies, the same metabolite chemical structures were referenced. For all the unknown parameters, we substituted a single representative value, obtained by averaging across all known corresponding parameters.

**Figure 2:**
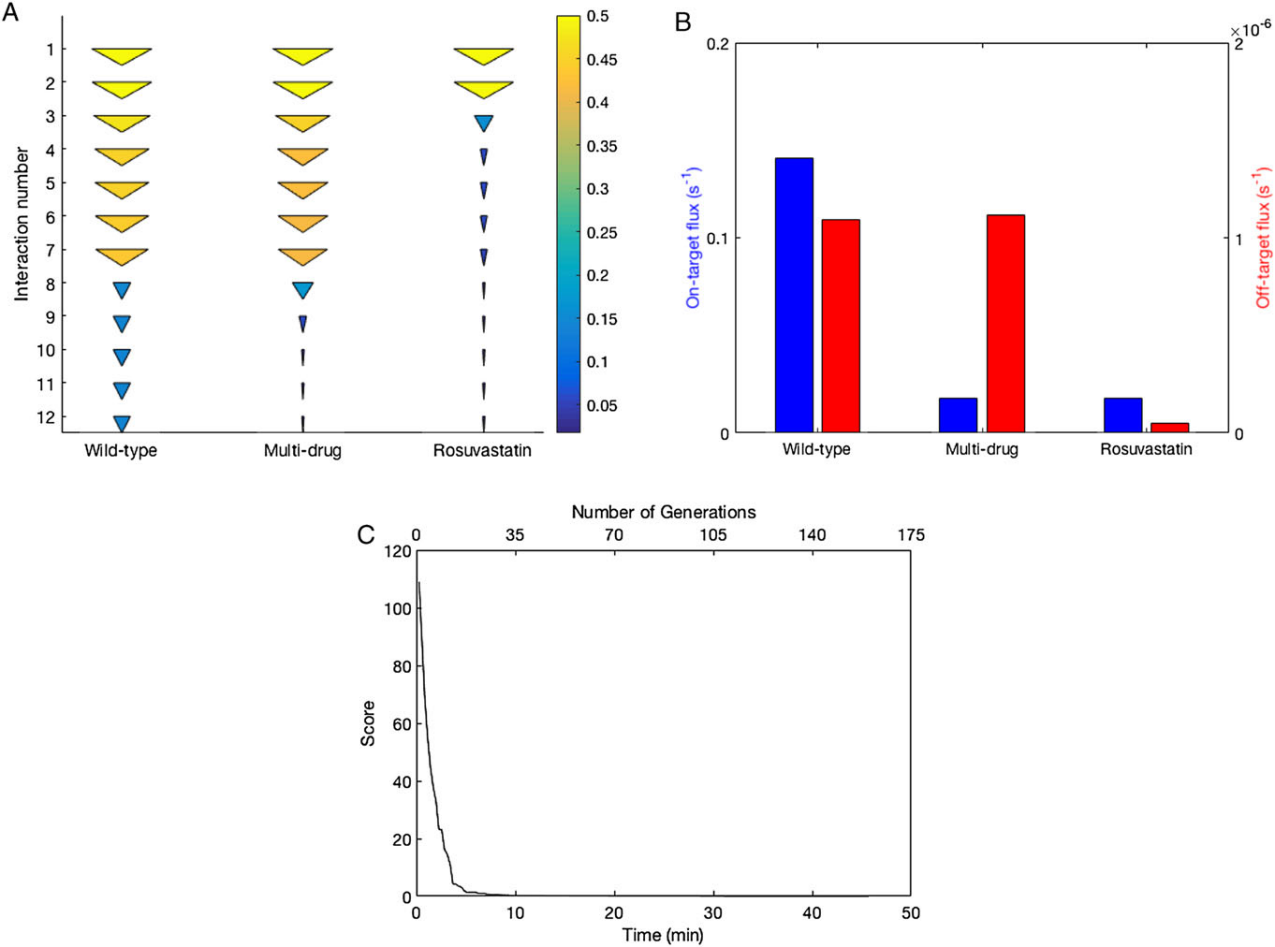
(A) The profile of flux through the pathway shown in Figure 1 described as a cone plot for the three scenarios: wild‐type (treatment free), optimized multi‐drug intervention and single‐drug statin‐like intervention. Cone size and colour both represent flux level. We show only the flux leading to cholesterol synthesis [the flux to protein prenylation is presented in (B)]. Interactions are numbered by their product: (1: 3‐hydroxy‐3‐methylglutaryl‐CoA; 2: melvaldyl‐CoA, 3: mevalonate, 4: mevalonate‐P, 5: mevalonate diphosphate, 6: isopentenyl diphosphate, 7: dimethylallyl diphosphate, 8: geranyl diphosphate, 9: farnesyl diphosphate, 10: presqualene diphosphate, 11: squalene, 12: cholesterol synthesis). (B) The flux through the endpoints of the two branches for the three scenarios: wild‐type, optimized multi‐drug intervention and single‐drug statin‐like intervention. Flux through the squalene/cholesterol synthesis branch is shown in blue. Flux through the geranylgeranyl‐PP/protein prenylation branch is shown in red. The statin concentration has been selected to ensure that the flux through the cholesterol synthesis branch is the same as in the multi‐drug intervention. (C) Convergence on the optimal multi‐drug intervention that suppresses cholesterol synthesis whilst minimizing off target effects, shown against time and against generations of the genetic algorithm.

Calculating the steady‐state behaviour of the pathway in the absence of inhibitors yielded the profile of flux shown on the left of Figure 2A, which we take to be wild‐type behaviour. Using computational optimization, we identified the following drug combination that produced the steady‐state profile of flux shown in the middle of Figure 2A and in Figure 2B: L‐659,699 = 0.0294 nM, rosuvastatin = 2.60 nM, farnesyl thiodiphosphate = 0.0340 nM, cinnamic acid = 0.00104 nM, 6‐fluoromevalonate 5‐diphosphate = 0.0213 nM, zoledronic acid = 9.97 nM, BPH‐628 = 5.86 nM; zaragozic acid A = 0.755 nM (see Table 3 and Supporting Information Tables S2 and S3). Here, the production of squalene, a precursor of cholesterol, is heavily suppressed and the production of geranylgeranyl diphosphate is maintained at wild‐type levels. In Figure 2B, we see specifically the flux at endpoints of the two pathway branches. With this drug combination, the flux from geranylgeranyl diphosphate → protein prenylation is the same between the wild‐type (inhibitor free) case and the optimal multi‐drug intervention case. However, the flux from squalene → cholesterol synthesis has been significantly suppressed.

In Figure 2A, B, we compare the flux profiles for wild‐type and optimal multi‐drug interventions to the case where rosuvastatin, a type of statin, is applied alone. This inhibitor targets the interaction catalysed by HMGCR, and we chose a concentration sufficient to suppress the rate of squalene formation and consumption to the same extent as the multi‐drug intervention. As can be seen in Figure 2B, rosuvastatin intervention impacts upon both branches of the pathway, suppressing geranylgeranyl diphosphate formation and protein prenylation as an off‐target effect of treatment.

Interestingly, a concentration of 362 nM rosuvastatin was required to achieve the same level of squalene suppression as the multi‐drug intervention. The greatest individual drug concentration required in the optimal multi‐drug intervention was 9.97 nM, and the total combined concentration was 19.3 nM, a dramatically lower dosage.

The value of drug combinations can also be seen in Supporting Information Figure S1 where we consider the impact of pairs of drugs (Lehár *et al.,* 2007). Here, we see that drug pairs with targets above the fork inhibit the flux through both pathway endpoints (Supporting Information Figure S1A, B). Drug pairs with targets above and below the fork together inhibit the flux through the cholesterol synthesizing branch (Supporting Information Figure S1C, D). However, drug pairs with targets above and below the fork at high doses can have a low impact on the flux through the protein prenylation branch (Supporting Information Figure S1E, F). Critically, Supporting Information Figure S1B, E shows that concentrations can be selected that significantly suppress the cholesterol synthesizing branch but that do not suppress the protein prenylation branch. The results demonstrate comparable impact to the multi‐drug intervention described above, but at higher individual and combined concentrations.

In order to identify the optimal multi‐drug combination, it was necessary to use a high‐performance computing (HPC) platform. However, the HPC demands were modest. Using an eight‐node desktop computer running MATLAB in parallel, we can see that the score (a dimensionless value, greater than or equal to zero, that quantifies how effectively the best performing multi‐drug intervention identified achieves our objective, with zero indicating success) converges rapidly on an effective drug combination. It successfully identified an optimal combination in 46 min and achieved an approximately optimal solution in less than 10 min.

The results of our curation of the pathway and the inhibitors that target it are available in GtoPdb at http://www.guidetopharmacology.org/GRAC/FamilyDisplayForward?familyId=104, an example of which is shown in Supporting Information Figure S2.

The model produced is available from http://biomodels.org (Chelliah *et al.,* 2013) (ID: MODEL1506220000).

## Discussion

### The importance of systems pharmacology

#### Multi‐drug interventions

Multi‐drug approaches are already employed in areas including HIV and oncology (Petrelli and Giordano, 2008; Thakur and Marchand, 2012). However, the existing interventions have typically been developed heuristically, rather than through systematic studies of the pathways involved, requiring significant domain expertise and subjective judgement. Systems pharmacology introduces objective metrics that have the potential to transform therapy development, yielding therapeutic hypotheses more rapidly and cost‐effectively.

Many diseases are multifactorial in nature, involving multiple pathways in their pathology. Effective future therapies will likely employ multi‐drug approaches that target multiple points in the network of pathways responsible (i.e. polypharmacology). Promiscuous drugs can be incorporated advantageously into the generation of these hypothetical interventions, provided that their interactions are known and parameterized.

Multi‐drug approaches can minimize the pleotropic effects of an intervention. As we demonstrated for statins, where a single drug intervention suppressed the output of a pathway to the same extent as multiple drugs targeting the same pathway, not only was the dose of each of the multiple drugs significantly lower than the dose of the single drug but also the combined dose of all of the multiple drugs was significantly lower than the dose of the single drug. This intrinsically reduces the risk from off‐target or pleotropic effects for each drug.

The systems pharmacology approach allows us to predict multi‐drug strategies that may be optimal to treat a disease and can be used as a prioritization triage for future drug development. It can support personalized and stratified medicine, where we adapt the parameter sets of the underlying models of pathway activity to represent an individual (for personalized medicine) or a subpopulation (for stratified medicine) and we develop interventions that are customized to be optimal for the patient or patient group. A challenge lies in developing optimized therapies so that they preferentially target key tissues. Pathway models and pharmacological interactions can be made tissue specific by generating a new parameter set for each tissue. Hypothesis generation would then use optimization to determine an intervention that impacted upon a key pathway in a key tissue, leaving other pathways unchanged across all tissues and with a minimal impact on the key pathway in non‐targeted tissues.

#### Drug development

Few multi‐drug treatments make it through the development process. The number of combinational therapies listed in the Therapeutic Target Database at the time of writing is 115 (Qin *et al.,* 2014). A combination therapy, LCZ696, with the brand name *Entresto*, was approved in 2015 and is in Phase III of clinical trials for the treatment of cardiovascular disorders. Establishing drug combinations using a conventional drug development pipeline creates significant challenges as development essentially replicates the single drug development process multiple times. Systems pharmacology is therefore critical to expanding the range of multi‐drug interventions available in a cost‐effective manner. Although it may add extra steps to the preclinical stages of the drug development process, it could have a significant positive impact on the cost‐efficiency associated with each success by reducing the attrition rate in the later stages of the pipeline (Bowes *et al.,* 2012).

Integrating our understanding of pharmacology and systems biology will also enable us to make better predictions of the behaviour of individual drugs. For example, squalene synthase (FDFT1) has been investigated as a potential drug target that lies downstream of HMGCR, the target for statins, in the cholesterol biosynthesis pathway (see Figure 1). FDFT1 catalyses an interaction after the fork to geranylgeranyl‐diphosphate production, and it has been speculated that squalene synthase inhibitors might suppress cholesterol production without impacting on the geranylgeranyl‐diphosphate producing branch, in contrast to statin treatment. However, squalene synthase inhibitors typically have K_i_ values orders of magnitude greater than the typical K_i_ for statins (See Table 3). As a result, squalene synthase inhibitor concentrations are required to be orders of magnitude greater than statin concentrations to suppress the corresponding enzyme activity comparably. Such high concentrations risk unforeseen off‐target effects, making squalene synthase inhibitors a higher risk drug to develop.

#### Systems level analysis

At the heart of systems pharmacology is the growing recognition that we will only be able to truly understand the best ways to therapeutically intervene in physiological function by considering biology at a systems level. The network of interactions that mediate physiological function is a dynamical system, and just as health and disease are different dynamical states of cells, tissues and organs, they also describe different dynamical states of the networks (Ahn *et al.,* 2006). In a network context, dynamical states can comprise a single stable configuration of the whole network or a sequence of configurations that repeat cyclically and stably. However, it is the configuration (species concentrations, distributions and structural conformations) of the network as a whole, or at least of critical subnetworks, that relate to phenotype, rather than any single component of the network (Lewis and Glass, 1991).

Small networks often yield dynamics that are intuitive and predictable. However, as networks become larger and richer in structure, novel and often counter‐intuitive dynamics can emerge and it will only be once we are able to build high‐confidence models at this scale that the full potential of systems level studies will be realized (Aderem, 2005). Building high confidence networks at this scale is inherently challenging as we see here. Coherently and unambiguously parameterizing all the interactions of a network is a significant logistical challenge. However, we have also seen that doing so enables us to identify and address the side‐effects of treatment whilst the therapy is being designed, rather than retroactively. Hence, systems‐level approaches are well suited to pharmacological applications.

### Current impediments to systems pharmacology

### Problem 1: lack of systematic recording

The absence of systematic and rigorous descriptions of metabolites and pharmaceutical compounds poses a significant challenge. Example 1, fluvastatin consists of two enantiomers, represented by PubChem compound identifiers (CIDs) 1548972 and 446155, with the 3R, 5S enantiomer (CID 446155) being significantly more pharmacologically active than the other (Di Pietro *et al.,* 2006; Boralli *et al.,* 2009). Commercial preparations used *in vitro* often vary in their stereochemical composition, with both enantiomers available individually, as well as in a racemic mixture. However, the authors did not always specify the stereochemical composition used despite this necessarily impacting upon the inhibition constant, K_i_, reported. Example 2, mevalonate is a metabolite that occurs naturally in mammals as the (R)‐isomer form. Sigma‐Aldrich currently refers to its marketed version as ‘(RS)‐mevalonic acid’. However, in one study (Potter and Miziorko, 1997), the metabolite is obtained from the supplier Sigma‐Aldrich, and it is recorded on BRENDA under the general name ‘mevalonic acid’ without unambiguous chemical identifiers such as the Simplified Molecular‐Input Line‐Entry System or the International Chemical Identifier. The isomer form affects the parameterization of the metabolite. Hence, this ambiguity creates potential inaccuracy in any resulting modelling.

### Problem 2: reporting of the wrong data

We found cases of incorrect or incomplete kinetic data reported in the primary literature that undermined the ability to model interactions. V_max_ values were regularly reported instead of K_cat_ values where V_max_ is related to K_cat_ by V_max_ = K_cat_ × (enzyme concentration). For a V_max_ value to be reusable in subsequent studies, the enzyme concentration must also be reported alongside it. However, we regularly found this not to be the case, making most reported V_max_ values unusable.

Similarly, inhibitors were frequently parameterized by IC_50_ values instead of K_i_ values, where K_i_ and IC_50_ are related by K_i_ = IC_50_/(1 + S/K_m_) and S is the substrate concentration. For IC_50_ values to be reusable in future studies, the substrate concentrations must also be reported. Here, again, we found regular omissions that rendered most reported IC_50_ values unusable.

#### Solution (1 and 2): introduce data capture standards that facilitate unambiguous reconstruction of the results without optimization

Reporting must include clear and thorough descriptions of experimental configurations and unambiguous identification of chemical structures through the use of comprehensive and standard nomenclature. Past experience has shown that effective standards can be developed through open community exercises (e.g. SBML and SBGN). The necessity for appropriate standards has been recognized previously by the chemical biology and pharmacometric communities (Oprea *et al.,* 2011; Swat *et al.,* 2015).

Standards are already employed widely across the life sciences, frequently building upon ontologies (controlled vocabularies of biological/chemical entities and concepts). The International Union of Pure and Applied Chemistry, the International Union of Biochemistry and Molecular Biology (IUBMB) Joint Commission on Biochemical Nomenclature and the Nomenclature Committee of IUBMB have provided guidelines on biochemical descriptions and enzyme classification. A library of ontologies for the life sciences has also been proposed by the Open Biomedical Ontologies Foundry (Smith *et al.,* 2007). Standards and guidelines also exist for reporting biomedical studies, including the minimum information (MI) standards overseen by the Minimum Information for Biological and Biomedical Investigations Foundry who include the Standards for Reporting Enzymology Data Commission (Gardossi *et al.,* 2010). The MI standards of direct relevance include the ‘minimum information about a bioactive entity’ (Orchard *et al.,* 2011), the ‘minimum information about a proteomic experiment’ (Taylor *et al.,* 2007) and the ‘minimum information about a molecular interaction experiment’ (Orchard *et al.,* 2007).

### Problem 3: curation errors

Online databases can contain errors. We have identified cases where the incorrect structures, enzyme targets, species and parameter values had been recorded. Errors were at low frequency, but some would undermine systems pharmacology approaches, and these fell into two groups: errors that derived from mistakes in the literature itself, such as from mis‐interpretation of data, and errors that derived from the incorrect transcribing from the literature to the database. The former derive from verbatim acceptance of results from manuscripts following author error. The latter errors can be introduced by databases themselves, either from semi‐automated triage tools or inadvertent curator mistakes, and this can be associated with a lack of clarity in the original document. In the present study and for the GtoPdb, we reviewed the primary literature when expanding our datasets and re‐curated existing database coverage.

#### Solution 3: quality control in curation of results

Using teams of curators to validate each other's work can reduce errors. This can be arranged systematically into error‐identifying or error‐correcting curation quality control programmes. In an error‐identifying programme, each result is independently curated twice and where disagreements are identified, the data is reviewed. Such approaches have been discussed within the International Society for Biocuration (Bateman, 2010). However, the funding limitations of most public databases preclude this degree of validation. In an error‐correcting programme, each result would be independently curated three times and where a disagreement is found, the consensus would be accepted automatically as correct.

### Systems pharmacology for the future

#### A workflow for future studies and hypothesis generation

With an adequate set of standards and a well‐characterized experimental system, it should be possible to develop intervention hypotheses that can be tested to inform future therapy development and to contribute to iterative refinement of databases. To make this a consistent, high confidence process, it would be advantageous to work in one experimental system. Such an experimental system could be *in vivo* or *in vitro*. However, an *in vitro* model would offer more control and consistency. Such an *in vitro* system would serve as a first approximation to *in vivo* physiology and would contribute to determining how *in vitro* parameters are mapped to *in vivo* parameters in order to maximize their value. An advantage of using an *in vitro* system is that it would lend itself to automated hypothesis generation and testing and it could be used to systematically search for new protein–protein and drug–target interactions. It has been suggested that artificial intelligence methods would be suitable for this purpose in the laboratory (King *et al.,* 2004). Automation would both minimize the time required for study and reduce the risk of misreporting or mis‐curating the results.

Our current systems‐level understanding has grown to a scale where manual manipulation is no longer feasible. Standards such as SBML, SBGN and SBGN‐ML and repositories such as BioModels have been developed partially to address this and automated model development allows the full value of databases to be realized (Swainston *et al.,* 2011). Open Pharmacological Concept Triple Store (Williams *et al.,* 2012) is a consortium responsible for a number of pharmacological and life science databases whose aims include the improvement of data availability through the use of data standards, the incorporation of contextual data through semantic web standards and the cross‐platform linkage of datasets through an identity mapping service. Developing multi‐drug hypotheses is a challenge that grows exponentially with the number of drugs and interactions considered. HPC resources are likely to be essential for this development.

**Figure 3:**
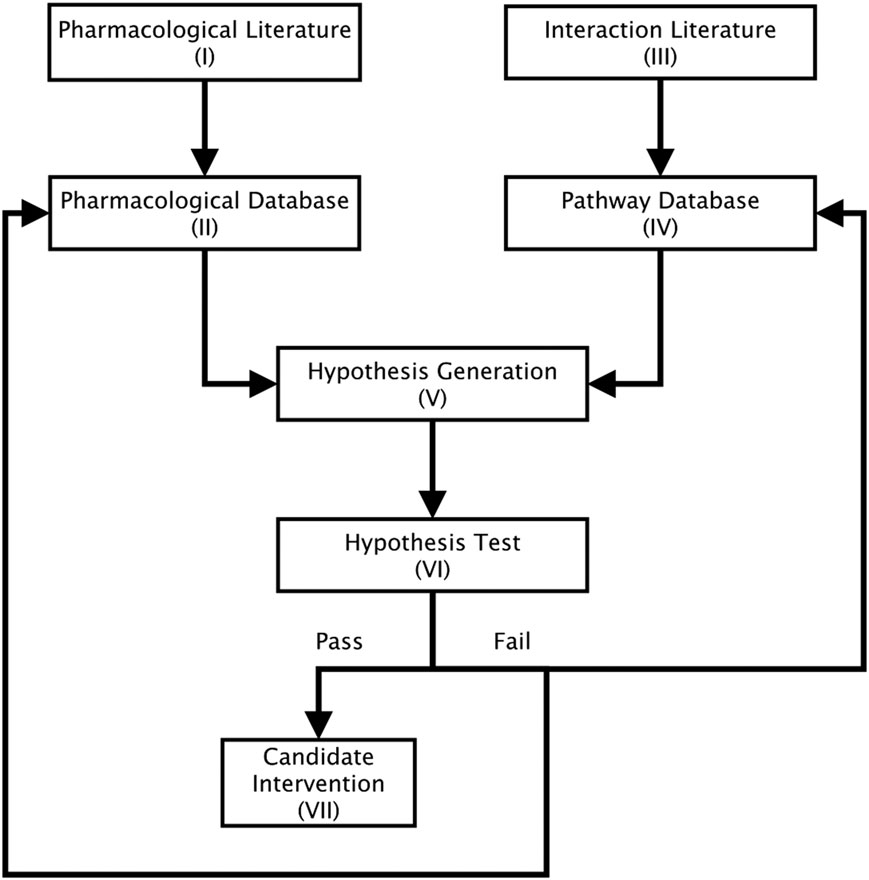
The proposed systems pharmacology workflow.

The following workflow would enable the process to be automated (see Figure 3).

- Pharmacological literature seeds databases of pharmacological interactions.
- Pharmacological and chemical databases containing sufficient information for experimental results to be reproduced accurately. Database Application Program Interfaces (APIs) facilitate extraction of results for hypothesis generation.
- Interaction literature seeds databases of biological pathways.
- Pathway databases containing sufficient information for experimental results to be reproduced accurately. Database APIs facilitate extraction of results for hypothesis generation.
- Hypothesis generation for single drug and multi‐drug interventions using data obtained through APIs from the pharmacological and pathway databases.
- Hypothesis testing. Success yields a candidate therapy and provides validation of the database. Failure initiates further exploration of the underlying interactions that in turn refine the databases.
- Candidate Intervention. Following success, the group of compounds enters an optimization pipeline that reduces them to a minimal set of lead compounds for preclinical testing to establish their efficacy and safety.

## Conclusion

The growth in our understanding of pharmacological interactions and the continuing development of our ability to computationally model pathway biology will increasingly enable us to explore drug combinations that target multiple points on multiple pathways to reprogram system level behaviour. In this way, systems pharmacology may lead to more effective therapies with fewer side effects. Here, we explored this approach for the mevalonate arm of the cholesterol biosynthesis pathway, and in doing so, we identify many of the current barriers to progress.

We attempted to build a systems pharmacology model of the mevalonate arm of the cholesterol biosynthesis pathway, but gaps and inconsistencies in the data prevented us from achieving this to a high level of confidence. In particular, we found the lack of comprehensive and systematic parameterizations, experimental variation, ambiguity in structural detail and inappropriate and inaccurate reporting from the primary literature to be obstacles. That this should be the case for a pathway of such high biomedical and commercial significance was unexpected. For this reason, our best current parameterization represents a patchwork of values taken from multiple species and experimental configurations. Nonetheless, by completing gaps in our knowledge with representative values, we were able to demonstrate subtle reprogramming of pathway dynamics that may contribute significantly to drug development. We propose that these obstacles can be removed through the adoption of standards and quality control.

Although we focused on the mevalonate arm of cholesterol biosynthesis, this approach could be applied to any pathway of interest for which targets and ligands are known. However, before this can happen at a general level, both the computational biology and the pharmacology communities must collaborate to remove the current barriers to progress.

## Author contributions

This work was conceived by H.B., S.W., P.G. and A.H. The analysis and data compilation was undertaken by H.B., S.W., J.S. and A.P. The manuscript was written by H.B., S.W., J.S., C.M., A.P., C.S. and P.G.

## Conflict of interest

H.B., J.S., C.M., C.S. and A.H. have served as curators for the IUPHAR/BPS GtoPdb.

## Declaration of transparency and scientific rigour

This Declaration acknowledges that this paper adheres to the principles for transparent reporting and scientific rigour of preclinical research recommended by funding agencies, publishers and other organisations engaged with supporting research.

## Supporting information


**Figure S1** A sensitivity analysis of pairs of drugs within the pathway model with colour indicating the degree of pathway inhibition. Here we plot D = (1‐IF/WTF) where IF is the inhibited flux through the endpoint of the pathway and WTF is the wild‐type flux through the endpoint in the absence of inhibitors. Green indicates low inhibition (D = 0). Red indicates high inhibition (D = 1). With eight drugs there are seventy–two possible pairings. These six heat map plots have been selected as being representative of the results. The IC10, IC20, IC30, IC40, IC50, IC50, IC60, IC70, IC80 and IC90 were identified for each drug in isolation. We then combined pairs of drugs at these concentrations and evaluated their effects by calculating the resulting D value. Values of D with both drugs at IC10 are bottom left and with both drugs at IC90 are top right. For Rosuvastatin the IC10‐IC90 concentrations were (4.2, 10.9, 20.7, 35.1, 56.1, 87.7, 138.0, 228.3, 442.7) nM; for Farnesyl Thiodiphosphate the IC10‐IC90 concentrations were (325.4, 732.1, 1255, 1952, 2929, 4393, 6833, 11 716, 26 360) nM; for Cinnamic acid the IC10‐IC90 concentrations were (27829629.8, 62617146.82, 107344511.7, 166981644.3, 250474415.3, 375714567.5, 584449515.1, 1 001 921 461, 2 254 341 919) nM and for Zaragozic acid A the IC10‐IC50 concentrations were (0.5, 0.9, 1.3, 1.7, 2.1, 2.6, 3.3, 4.3, 6.4) nM.

**Figure S2** A representative reaction from the mevalonate arm of the cholesterol biosynthesis pathway, as described on the IUPHAR/BPS GuidetoPharmacology (GtoPdb).

**Table S1** The publicly available pathway and chemical databases used.

**Table S2** The inhibitors used in the model of the pathway with structural information. The inhibitors selected were those with the greatest efficacy in humans.

**Table S3** The ten best performing drug combinations identified using the genetic algorithm as part of hypothesis generation, together with their scores.

**File S1** Supplementary_Mevalonate_Pathway.sbgn A biologically meaningful, machine readable SBGN file encoding the diagram shown in Figure 1.

**File S2** Supplementary_Mevalonate_Pathway.sbml A biologically meaningful, machine readable SBML file encoding the mathematical model describing the pathway shown in Figure 1.
