# Supplementary figures and images for: Is systems pharmacology ready to impact upon therapy development? A study on the cholesterol biosynthesis pathway
Source: Br J Pharmacol. 2017 Nov 26;174(23):4362–82. doi: 10.1111/bph.14037 (PMC5715582; doi:10.1111/bph.14037)

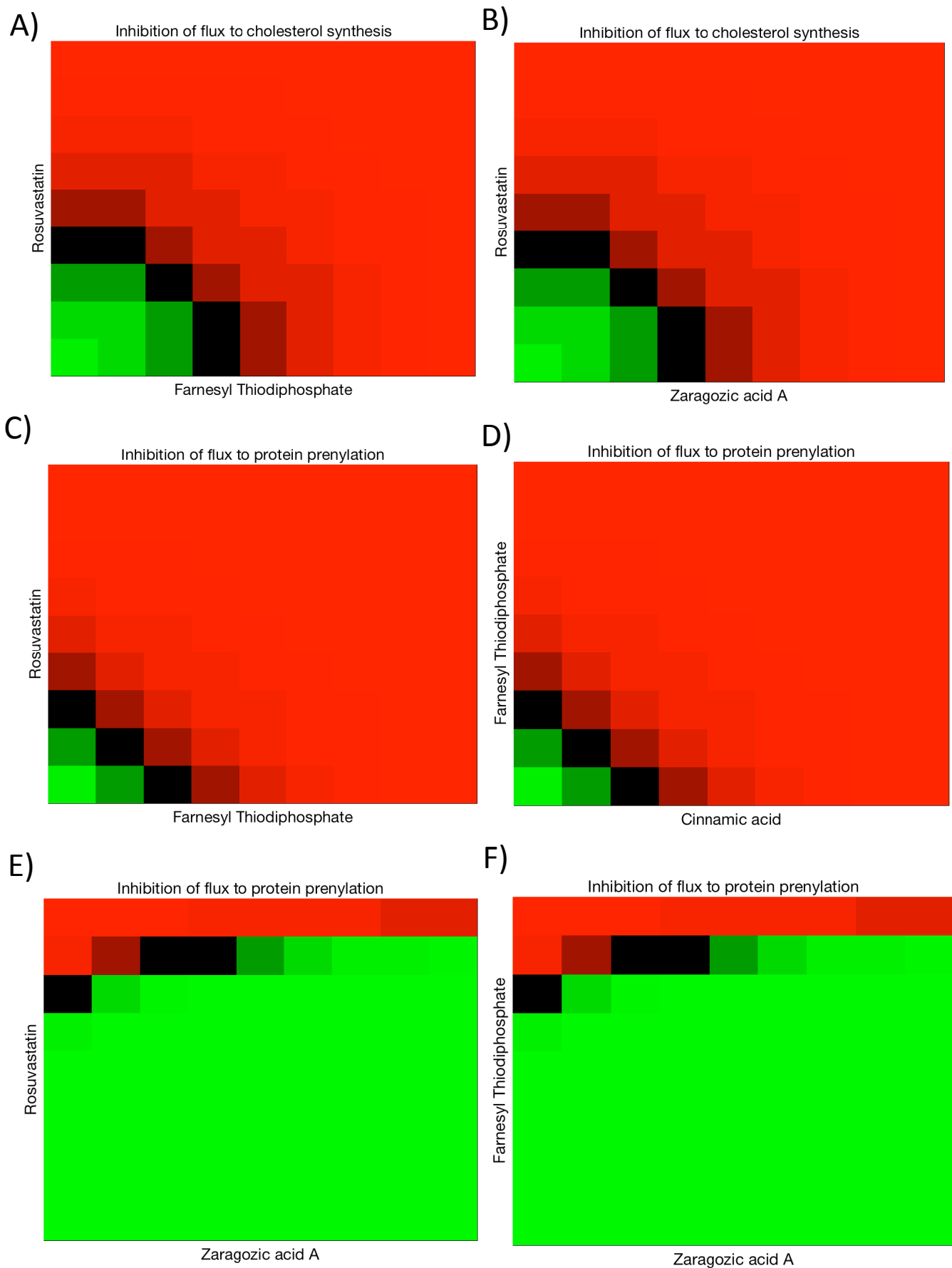

Supplementary Figure 1.

Supplement: Supplementary file 1 — Figure S1 A sensitivity analysis of pairs of drugs within the pathway model with colour indicating the degree of pathway inhibition. Here we plot D = (1‐IF/WTF) where IF is the inhibited flux through the endpoint of the pathway and WTF is the wild‐type flux through the endpoint in the absence of inhibitors. Green indicates low inhibition (D = 0). Red indicates high inhibition (D = 1). With eight drugs there are seventy–two possible pairings. These six heat map plots have been selected as being representative of the results. The IC10, IC20, IC30, IC40, IC50, IC50, IC60, IC70, IC80 and IC90 were identified for each drug in isolation. We then combined pairs of drugs at these concentrations and evaluated their effects by calculating the resulting D value. Values of D with both drugs at IC10 are bottom left and with both drugs at IC90 are top right. For Rosuvastatin the IC10‐IC90 concentrations were (4.2, 10.9, 20.7, 35.1, 56.1, 87.7, 138.0, 228.3, 442.7) nM; for Farnesyl Thiodiphosphate the IC10‐IC90 concentrations were (325.4, 732.1, 1255, 1952, 2929, 4393, 6833, 11 716, 26 360) nM; for Cinnamic acid the IC10‐IC90 concentrations were (27829629.8, 62617146.82, 107344511.7, 166981644.3, 250474415.3, 375714567.5, 584449515.1, 1 001 921 461, 2 254 341 919) nM and for Zaragozic acid A the IC10‐IC50 concentrations were (0.5, 0.9, 1.3, 1.7, 2.1, 2.6, 3.3, 4.3, 6.4) nM. [file BPH-174-4362-s001.pdf]
