# Supplementary material for: Is systems pharmacology ready to impact upon therapy development? A study on the cholesterol biosynthesis pathway
Source: Br J Pharmacol. 2017 Nov 26;174(23):4362–82. doi: 10.1111/bph.14037 (PMC5715582; doi:10.1111/bph.14037)
Supplement: Supplementary file 2 — Figure S2 A representative reaction from the mevalonate arm of the cholesterol biosynthesis pathway, as described on the IUPHAR/BPS GuidetoPharmacology (GtoPdb). [file BPH-174-4362-s002.pdf]

Enzyme Reaction

EC Number: 5.3.3.2	Isopentenyl diphosphate = dimethylallyl diphosphate

Substrates and Reaction Kinetics

Substrate		Sp.	Property	Value	Units	Assay description	Assay conditions	Comments	Reference
isopentenyl diphosphate	E	Hs	K_m	$3.3x10^{-5}$	M	Recombinant human protein expressed in E coli and purified	pH 7, 37°C	Km value obtained using the Grafit curve-fitting programme. 10-500 μM IPP; 20mM $MgCl_2$	2
isopentenyl diphosphate	E	Hs	V_{max}	4.1	μmol/min/mg	Recombinant human protein expressed in E coli and purified. 0.02μg of enzyme used in assay.	pH 7, 37°C	Vmax value obtained using the Grafit curve-fitting programme. 10-500 μM IPP; 20mM $MgCl_2$	2

Cofactors

Cofactor	Species	Comments	Reference
Mg^{2+}	Human	Enzyme activity increases with increasing Mg2+ up to 20mM.	2
Mn^{2+}	Human	Activity increases up to a concentration of 100 micromolar Mn2+ then sharply decreases.	2,5

Download all structure-activity data for this target as a CSV file GO

Inhibitors

Key to terms and symbols | View all chemical structures | Click column headers to sort

Ligand			Sp.	Action	Affinity	Units	Reference	
NE21650		S	Hs	Inhibition	~4.2	pIC_{50}	4	▼

Supplementary Figure 2.
