# Supplementary material for: Is systems pharmacology ready to impact upon therapy development? A study on the cholesterol biosynthesis pathway
Source: Br J Pharmacol. 2017 Nov 26;174(23):4362–82. doi: 10.1111/bph.14037 (PMC5715582; doi:10.1111/bph.14037)
Supplement: Supplementary file 3 — Table S1 The publicly available pathway and chemical databases used. [file BPH-174-4362-s003.pdf]

	Database/ Reference	Name of the pathway	URL	Cross pointers		
			Pathway URL	Ligands	Targets	Pathways
Pathway database	KEGG/[1]	Terpenoid backbone biosynthesis	http://www.genome.jp/dbget-bin/www_bget?map00900	PubChem, ChEBI, Lipidmaps, PDB, CAS number	ExploreEnz; IUBMB Enzyme nomenclature; ExPASy, BRENDA	n/a
	MetaCyc/[2]	Super pathway of cholesterol biosynthesis; Mevalonate pathway I	http://www.metacyc.org/META/NEW-IMAGE?type=PATHWAY&object=PWY66-5; http://www.metacyc.org/META/NEW-IMAGE?type=PATHWAY&object=PWY-922	CAS number; ChEBI, HMDB, KEGG, MetaboLights, PubChem	UniProt; Entrez	n/a
	Reactome/[3]	Cholesterol biosynthesis (*Homo sapiens*)	http://www.reactome.org/PathwayBrowser/#DIAGRAM=191273&PATH=1430728,556833	ChEBI	UniProt	n/a
	WikiPathways/[4]	Cholesterol biosynthesis (*Homo sapiens*)	http://www.wikipathways.org/index.php/Pathway:WP197	CAS	Entrez	n/a
			**Example entity URL**			
Enzyme database	Sabio RK/[5]	n/a	http://sabio.villa-bosch.de/reacdetails.jsp?reactid=2215	PubChem, ChEBI	UniProt	KeGG
	BRENDA/[6]	n/a	http://www.brenda-enzymes.info/enzyme.php?ecno=1.1.1.34&Suchword=&organism%5B%5D=Homo+sapiens&show_tm=0	n/a	ExplorEnz, ExPASy, KEGG, MetaCyc and others (all linking via EC number)	KeGG, Metacyc
Chemical database	ChEMBL/[7]	n/a	Ligand: https://www.ebi.ac.uk/chembl/compound/inspect/CHEMBL1078 Target: https://www.ebi.ac.uk/chembl/target/inspect/CHEMBL402	ChEBI, PubChem, Chemspider, Wikipedia	UniProt, Reactome, Ensembl	n/a
	PubChem/[8]	n/a	https://pubchem.ncbi.nlm.nih.gov/compound/1548972		n/a	n/a

Supplementary Table 1.
