# Supplementary material for: Is systems pharmacology ready to impact upon therapy development? A study on the cholesterol biosynthesis pathway
Source: Br J Pharmacol. 2017 Nov 26;174(23):4362–82. doi: 10.1111/bph.14037 (PMC5715582; doi:10.1111/bph.14037)
Supplement: Supplementary file 4 — Table S2 The inhibitors used in the model of the pathway with structural information. The inhibitors selected were those with the greatest efficacy in humans. [file BPH-174-4362-s004.pdf]

Enzyme	Inhibitor name	GTP Ligand ID	Isomeric SMILES	Ligand image	InChi Key
HMGCS1	L-659,699	5886	OC[C@H]1C(=O)O[C@@H]1CCCC[C@H](C/C(=C/C(=C/C(=O)O)/C)/C)C	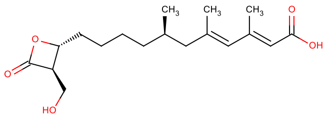	ODCZJZWSXPVLAW-KXCGKLMDSA-N
HMGCR	Rosuvastatin	2954	O[C@H](C[C@H](CC(=O)O)O)/C=C/c1c(nc(nc1c1ccc(cc1)F)N(S(=O)(=O)C)C)C(C)C	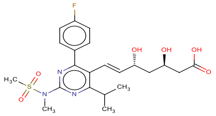	BPRHUIZQVSMCRT-YXWZHEERSA-N
MVK	Farnesyl Thiodiphosphate	3216	CC(=CCOP(=O)(OP(=O)(S)[O-])[O-])CCC=C(CCC=C(C)C)C	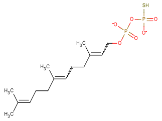	DRADWUUFBCYMDM-UHFFFAOYSA-L
PMVK	Cinnamic acid	3203	OC(=O)/C=C/c1ccccc1	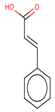	WBYWAXJHAXSJNI-VOTSOKGWSA-N
MVD	6-fluromevalonate 5-diphosphate	3205	FCC(OP(=O)(OP(=O)(O)O)O)CC(CC(=O)O)(O)C	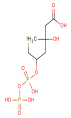	YGLNCOGHKIHKSA-UHFFFAOYSA-N
FDPS	zoledronic acid	3177	OP(=O)(C(P(=O)(O)O)(Cn1cncc1)O)O	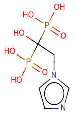	XRASPMIURGNCCH-UHFFFAOYSA-N
GGPS1	BPH-628	3188	OP(=O)(C(P(=O)(O)O)(Cc1cccc(c1)c1ccc(cc1)c1ccccc1)O)O	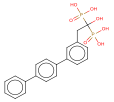	MPBUFKZCEBTBSK-UHFFFAOYSA-N
FDFT1	zaragozic acid A	3057	CC[C@@H](C[C@@H](/C=C/C(=O)O[C@@H]1[C@@H](O)[C@@]2(O[C@@]1(C(=O)O)[C@](O)(C(=O)O)[C@H](O2)C(=O)O)CCC(=C)[C@H]([C@@H](Cc1ccccc1)C)OC(=O)C)C)C	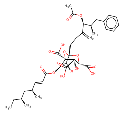	DFKDOZMCHOGOBR-NCSQYGPNSA-N

Supplementary Table 2.
