# Supplementary material for: Is systems pharmacology ready to impact upon therapy development? A study on the cholesterol biosynthesis pathway
Source: Br J Pharmacol. 2017 Nov 26;174(23):4362–82. doi: 10.1111/bph.14037 (PMC5715582; doi:10.1111/bph.14037)
Supplement: Supplementary file 5 — Table S3 The ten best performing drug combinations identified using the genetic algorithm as part of hypothesis generation, together with their scores. [file BPH-174-4362-s005.pdf]

Solution Number	L-659,699	Rosuvastatin	Farnesyl Thiodiphosphate	Cinnamic acid	6-fluromevalonate 5-diphosphate	zoledronic acid	BPH-628	Zaragozic acid	Scores
1	0.029447	2.600692	0.034037	0.001042	0.021293	9.966505	5.861416	0.755219	0
2	0.029447	2.600692	0.037943	0.005254	0.021293	9.965849	5.860653	0.755219	2.8966E-07
3	0.033354	2.600692	0.037943	0.001042	0.021293	9.966627	5.860653	0.755219	9.6048E-07
4	0.029447	2.600692	0.037943	0.001042	0.021293	9.965181	5.860287	0.754898	1.1646E-06
5	0.029447	2.600692	0.037943	0.004743	0.021293	9.966505	5.860653	0.755219	1.1973E-06
6	0.029447	2.602645	0.037943	0.001042	0.021293	9.966505	5.860653	0.755219	1.7037E-06
7	0.029447	2.600692	0.037943	0.001042	0.021293	9.966627	5.860653	0.755219	3.4526E-06
8	0.029447	2.600692	0.037943	0.004743	0.030082	9.966505	5.860653	0.755219	3.5275E-06
9	0.029447	2.600692	0.037943	0.001042	0.021293	9.971449	5.860653	0.755219	3.9832E-06
10	0.029447	2.600692	0.037943	0.001042	0.021293	9.968763	5.860653	0.755219	4.7565E-06

Supplementary Table 3
